# Determinants of residual myometrial thickness after cesarean delivery: Comparative analysis of barbed versus conventional sutures—A sub‐analysis from the SPIRAL trial

**DOI:** 10.1002/ijgo.70273

**Published:** 2025-06-05

**Authors:** Jota Maki, Hikaru Ooba, Tomohiro Mitoma, Hikari Nakato, Ayano Suemori, Chiaki Kuriyama, Shujiro Sakata, Sakurako Mishima, Akiko Ohira, Eriko Eto, Hisashi Masuyama

**Affiliations:** ^1^ Department of Obstetrics and Gynecology Okayama University Graduate School of Medicine, Dentistry and Pharmaceutical Sciences Okayama Japan

**Keywords:** barbed suture, cervical ripening, cesarean scar defect, cesarean scar disorder, niche, residual myometrial thickness, risk factors

## Abstract

**Objective:**

This sub‐analysis aimed to determine whether conventional suture‐associated risk factors for cesarean scar defect show similar outcomes with barbed continuous suturing, and to identify factors influencing residual myometrial thickness when using barbed continuous sutures.

**Methods:**

This sub‐analysis of a multicenter, parallel‐group, randomized controlled trial across four Japanese obstetrics and gynecology departments included 1211 women who had their first cesarean delivery between May 2020 and March 2023. Among them, 298 women underwent a C‐section, with 253 follow‐up through July 2023. Singleton pregnancies were randomly assigned to receive either barbed or conventional double‐layered continuous sutures in a 1:1 ratio; they were monitored from consent through their 6‐ to 7‐month check‐up. The effects of cervical ripening, facility characteristics, and surgeon experience were investigated using a two‐way ANOVA.

**Results:**

Of the remaining 253 patients, 33 were lost to follow‐up and 220 completed follow‐up (110 per group). One institution enrolled the largest proportion of participants (45.9%), whereas two other institutions had more experienced surgeons. Two‐way ANOVA revealed that surgeon experience (*P* = 0.020) and institutional factors (*P* < 0.001) significantly influenced the residual myometrial thickness at 6–7 months after surgery, whereas cervical dilation during active labor did not (*P* = 0.215). Additionally, a significant interaction was observed between institutional factors and suture type (barbed vs. conventional) on residual myometrial thickness (*P*_interaction_ <0.001).

**Conclusion:**

Institutional factors and surgeon experience represent significant determinants of residual myometrial thickness when using barbed sutures for cesarean closure, highlighting the importance of standardized surgical protocols and training across facilities.

## 1 INTRODUCTION

Cesarean sections account for approximately 25%–40% of deliveries in Japan,^1^ resulting in concerns about cesarean scar defects (CSDs).^2^, ^3^ About 30% of cases in which CSD is recognized, cesarean scar disorder can lead to muscular defects at the scar site, potential infertility, future pregnancy complications, and increased menstrual bleeding.^4^, ^5^, ^6^ The degree of myometrial thinning may vary depending on several suturing factors, including continuous versus interrupted sutures,^7^ single‐ versus double‐layer closure,^8^ whether the decidua is included in the closure,^9^ and the type of suture material used. However, uterine incision closure techniques vary by region and institution. Additionally, the surgeon's level of expertise significantly influences the extent of CSD.^7^ For cesarean sections, surgeons are recommended to perform 10–40 procedures under supervision before operating independently.^10^

While efforts to address cesarean scar disorder using advanced suturing techniques continue to evolve,^9^, ^11^ recent studies have advocated the advantages of barbed sutures, which self‐anchor without knot‐tying while ensuring tissue alignment.^12^, ^13^, ^14^ These findings underscore the importance of preventing CSD and suggest that myometrial suturing using barbed sutures might be useful. Barbed sutures offer close adherence to the muscle layer and exhibit superior hemostatic capabilities.^15^ Their design ensures wound closure without undue tension and avoids suture failures stemming from absent knots,^13^, ^16^ thus making them suitable for organs with expansive tendencies.^17^ We previously conducted a randomized clinical trial comparing barbed sutures with conventional sutures in cesarean section patients across four obstetric departments spanning three medical regions in Japan.^18^ The results demonstrated that, compared with double‐layer continuous suturing, continuous barbed suturing significantly improved niche measurements (length, depth, and width; mean scores 2.45 versus 3.79 mm [*P* < 0.001], 1.78 versus 2.70 mm [*P* < 0.001], and 1.58 versus 2.88 mm [*P* < 0.001], respectively) and residual myometrial thickness (RMT; 8.46 versus 7.07 mm [*P* < 0.001]).^19^

Building on these promising results, this sub‐analysis aimed to determine whether previously reported risk factors for CSD with conventional sutures showed similar outcomes to those of barbed continuous sutures, and to identify factors influencing RMT when using barbed continuous sutures.

## 2 MATERIALS AND METHODS

### 2.1 Trial design

Our study objectives and protocol have been previously detailed (Data S1).^19^ Briefly, we employed a multi‐center, parallel‐group, 1:1 randomized controlled trial design. The study was conducted across four obstetrics and gynecology departments in three medical regions of Japan, encompassing primary to tertiary care facilities. The classification of the years of experience among the surgeons is as follows: 1–5 years, not yet certified by the Japanese Society of Obstetrics and Gynecology (JSOG); 6–15 years, certified by the JSOG and with 10 years of clinical experience; and 16 years or more, certified by the JSOG with more than 10 years of clinical experience. Consistent with SPIRIT guidelines, this randomized controlled trial adhered to the CONSORT 2010 checklist (Data S2 and S3). The study was approved by the Okayama University Certified Review Board (CRB19‐006) on April 9, 2020, and was registered with the Japan Registry of Clinical Trials (jRCT1062200001) on May 1, 2020 (Data S4 and S5). Further details are provided in Data S1.

### 2.2 Patient selection

The study enrolled pregnant women aged 20 years or older without prior cesarean sections who were referred to the obstetrics departments of any of the four participating hospitals. Participants were recruited before the cesarean section was deemed necessary, and written informed consent was obtained. The actual first registration date was May 7, 2020, and the final registration deadline was March 31, 2023. The inclusion and exclusion criteria are listed in Data S1.

### 2.3 Intervention

Post‐enrollment, eligible participants were randomly assigned into two equal groups, receiving either conventional sutures or barbed sutures. Each participant was assigned a unique identification code. During the cesarean section, a transverse incision measuring approximately 10 cm in length was made in the lower uterine segment. Following delivery, uterine repair was performed using either the allocated barbed sutures or conventional sutures. To ensure procedural consistency across institutions, the principal investigator demonstrated the technique at each facility. A comprehensive procedural manual was developed and distributed to all participating institutions using surgical videos and simulator‐based training. Although the medical staff were aware of the suture type, participants remained blinded. Details regarding the suturing technique and transvaginal ultrasound procedure for follow‐up have been previously published (Data S1).^15^, ^16^

### 2.4 Medical equipment used for the intervention

For the barbed thread, 0‐STRATAFIX® Spiral PDS Plus, which comprises 0.40‐ to 0.499‐mm polydioxanone sutures (Ethicon; Johnson & Johnson, New Brunswick, NJ, USA; JMDN code: 16584000; approval number: 22900BZX00123000) was utilized. In addition, 0‐Vicryl (Ethicon; Johnson & Johnson, New Brunswick, NJ, USA), which comprises 0.35‐ to 0.399‐mm polyglactin sutures (JMDN code: 17471000; approval number: 15700BZY01341000), was used for the conventional thread. The barbed thread was marginally thicker than its conventional counterpart to accommodate the barbs. Two suture layers were applied, with each layer consisting of a single suture. About 25% of the muscle tissue adjacent to the peritoneum (blood vessel layer) was left intact. The rest of the muscular tissue and internal lining were joined with continuous sutures to ensure proper alignment of the incision areas. While suturing the second layer, the terminal end was secured using either barbed or traditional suture material. The second suture layer completely covered the first layer's stitches, consistently incorporating the remaining quarter of muscle tissue on the serosal surface. The initial suture layer connected the inner lining and majority of muscle tissue, while the subsequent layer of sutures enveloped the first layer's stitches entirely.

### 2.5 Outcome assessment

A uterine niche was defined as an indentation ≥2 mm in depth at the cesarean section scar site, according to the modified Delphi criteria.^20^, ^21^ Scar defects were examined in both groups. The primary outcome was defined as the proportion of postoperative scar niches exceeding 2 mm in depth. The secondary outcomes included selected metrics such as surgery duration (from initiation to conclusion), time from childbirth to uterine closure completion, estimated blood loss, and the number of additional Z‐sutures required for hemostasis. Maternal complications during surgery, post‐surgical infections, and the occurrence of major complications were also monitored for safety assessment.

As these secondary outcomes have been comprehensively reported in our previous publication, we have provided only this abbreviated description to avoid redundancy while ensuring crucial elements are mentioned.^9^ Transvaginal ultrasonography was performed at 6–7 months postoperatively to evaluate uterine orientation and myometrial scar thickness. One or two experienced obstetricians (>1500 cases/year) at each facility conducted imaging evaluations using ultrasound systems (GE Medical Systems, Milwaukee, WI, USA; or Hitachi Aloka Medical, Tokyo, Japan) with a 7.5‐MHz center frequency transvaginal probe. The evaluating physicians received standardized training using reference materials and certification, following established guidelines. Scar niches were assessed using four transvaginal ultrasound measurements: (1) niche length, (2) niche depth, (3) RMT at the level of the deepest part, and (4) niche width (cross‐section). The niche size ratio (niche depth/[niche depth + RMT] × 100 [%]) and niche rate incidence (both niche length and depth ≥2 mm [%]) were calculated. All four measurements were compared between groups.^18^, ^19^

### 2.6 Sample size calculation

According to Zayed et al.,^22^ in their study “Barbed sutures versus conventional sutures for uterine closure at cesarean section,” detecting statistically significant differences in uterine closure time required 48 patients in each comparative group to achieve statistical parameters of 5% alpha error and 20% beta error. After accounting for an anticipated dropout rate of 5%, their protocol incorporated 50 patients in each cohort.

In a separate investigation utilizing conventional sutures, researchers documented a mean cesarean section scar thickness (our designated primary endpoint) of 4.18 mm with a standard deviation (SD) of ±1.76 mm.^23^ Based on our clinical experience with barbed sutures, we hypothesized a minimum enhancement in scar thickness of 1 mm compared with conventional suturing techniques. Furthermore, we reasonably anticipated that the standard deviation in our multicenter investigation would exceed that observed in single‐institution studies due to greater variability in surgical techniques and patient populations.

Employing Stata Statistical Software (Release 16; StataCorp LLC, College Station, TX, USA) for sample size calculation, we determined that 92 subjects per intervention group (totaling 184 participants) would be necessary to maintain the statistical significance level (*α*) at 0.05 while ensuring adequate statistical power (*β*) of 0.20. To compensate for an anticipated 10% participant attrition rate, we strategically increased the enrollment target to 260 participants (130 allocated to each comparative arm, sufficient to accommodate between 26 and 52 potential dropouts).^14^, ^24^

### 2.7 Analysis methods

The primary analytical focus was the full analysis set, encompassing all participants who received treatment during the study period. We employed *t*‐tests, the Mann–Whitney *U*‐test, or Fisher exact test to compare baseline patient data, ultrasonographic findings, adverse events, ultrasonographic abnormalities, and primary and secondary outcomes between the barbed and conventional suture groups. Cesarean scar dimensions were presented as quartile values. The primary efficacy outcome (scar dimensions) was compared between groups to determine the role of suture type in defect prevention.

This sub‐analysis specifically aimed to identify the influence of factors beyond suture comparison—cervical dilation status (≥6 cm), facility characteristics, and surgeons' years of clinical experience—on outcomes. We also examined the statistical interactions among these factors. All analyses were conducted using two‐way analysis of variance. Pairwise correlation analysis was performed to examine relationships between risk factors and scar niche parameters. The validity of the study and its analyses was verified by independent bodies: Okayama University of the Center for Innovative Clinical Medicine and the Department of Epidemiology. All statistical analyses were performed using SPSS Statistics (version 27.0; IBM Corp., Armonk, NY, USA), with a two‐sided *P* < 0.05 considered statistically significant.

## 3 RESULTS

**FIGURE 1:**
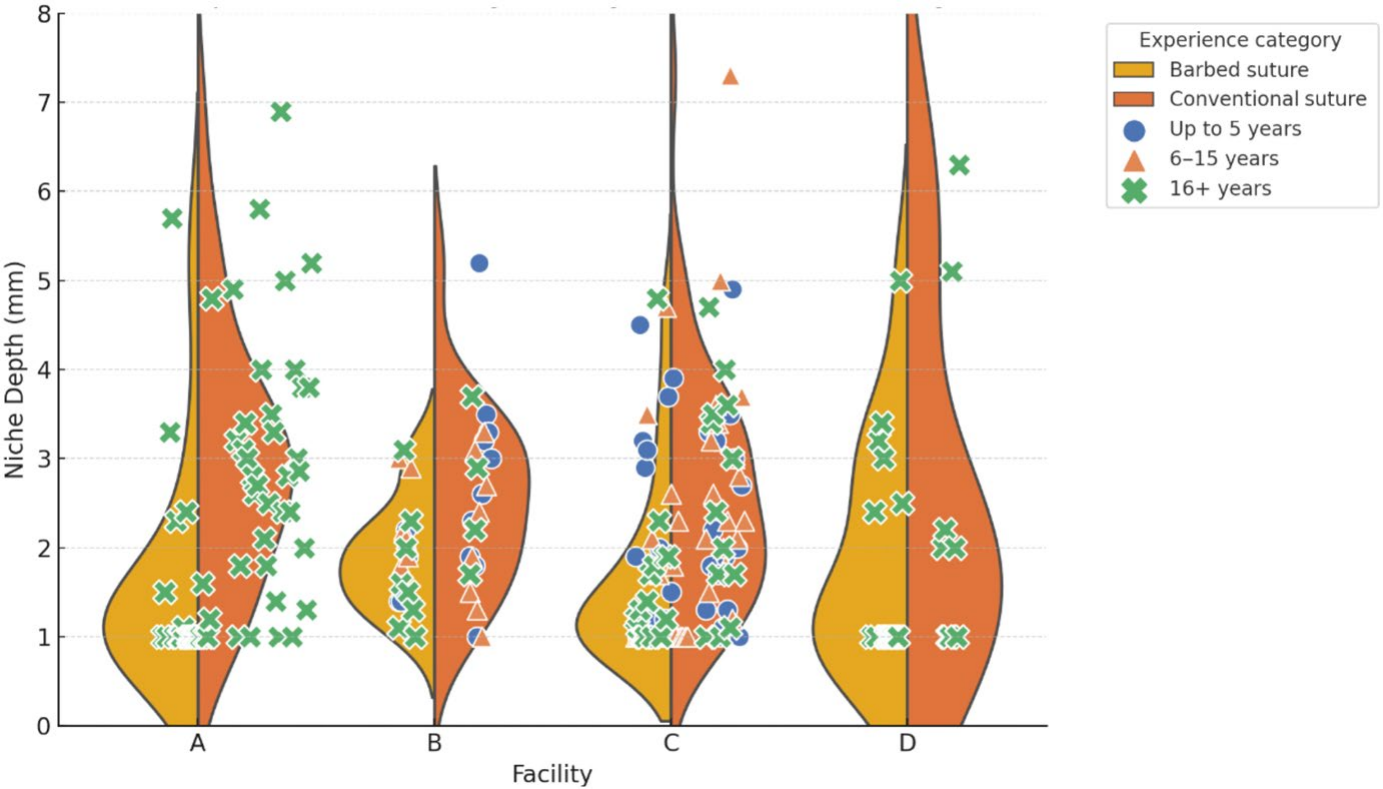
Density dot plot of cesarean scar defect distribution stratified by facility, suture type, and surgeon experience.

**FIGURE 2:**
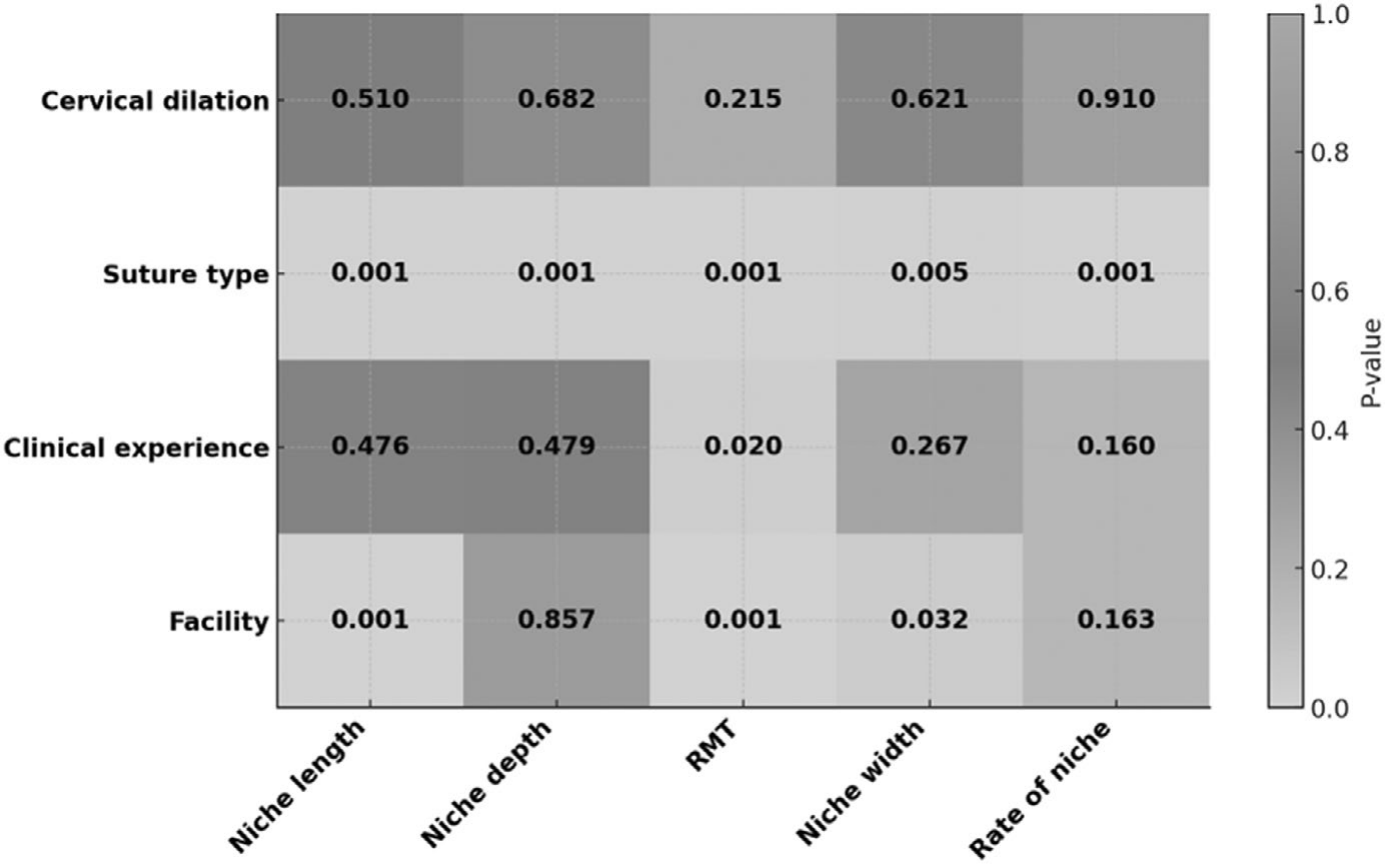
*P*‐values for factors affecting cesarean scar defect outcomes. RMT, residual myometrial thickness.

**FIGURE 3:**
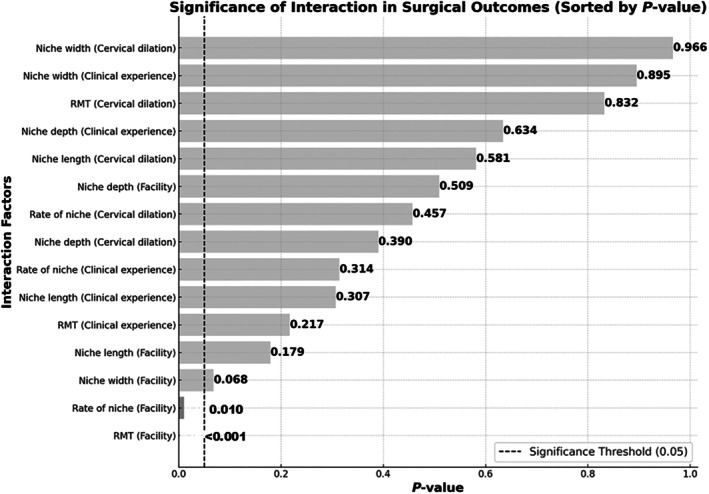
*P*‐values for interaction across risk factors. RMT, residual myometrial thickness.

The study concluded on March 31, 2024. Of the 253 patients, 33 were lost to follow‐up (barbed suture group, 17; conventional suture group, 16). Data for the remaining 220 participants (barbed suture group, 110; conventional suture group, 110) were available.^18^ Table 1 presents patient characteristics stratified by the four participating institutions. Institution C enrolled the largest proportion of participants (101 [45.9%]), whereas the surgeons at institutions A and D had more extensive clinical experience. Institutions B and C reported a higher proportion of cases with cervical dilation (≥6 cm). Table 1 and Figure 1 show the characteristics of each facility, surgical outcomes, and the distribution of RMT at 6–7 months post‐cesarean section, as well as the categorized years of experience of the surgeons. We analyzed background factors influencing this outcome. In addition to suture type being a risk factor (Figure 2; Table S1), both years of clinical experience and institutional factors showed significant effects (*P* = 0.020, *P* < 0.001). However, the degree of cervical dilation (≥6 cm) during active labor did not significantly impact the outcome (*P* = 0.215). Notably, the effect of barbed versus conventional suturing on RMT at 6–7 months post‐cesarean section showed significant institutional dependence (interaction test; *P*_interaction_ <0.001) (Figure 3; Table S1). Pairwise correlation analysis was performed to examine relationships between risk factors and CSD parameters. Analysis of suture type correlations revealed moderate positive associations with indentation depth (*r* = 0.355) and niche rate (*r* = 0.422). A moderate negative correlation between suture type and RMT (*r* = −0.336) was observed, while its relationship with cervical maturation was negligible (*r* = −0.041). Further analysis identified a weak negative correlation between surgical experience and RMT (*r* = −0.223), Facility‐specific variations were noted in both RMT (*r* = 0.204) and niche rate (*r* = −0.168), though these correlations were weak. The relationship between surgical experience and niche rate was minimal (*r* = 0.151). All other examined correlations yielded coefficients below *r* = 0.1, indicating no significant relationships between those parameters.

**TABLE 1:** Characteristics of facilities and surgical outcomes at 6–7 months of postoperative follow‐up.

Characteristic/facility (*n* [%])	Facility A (*n* = 56 [25.5])	Facility B (*n* = 41 [18.6])	Facility C (*n* = 101 [45.9])	Facility D (*n* = 22 [10.0])
Number of surgeons with clinical experience (*n* [%])				
1–5 years	0	5 (41.7)	6 (35.3)	0
6–15 years	0	4 (33.3)	8 (47.1)	0
16–39 years	2 (100.0)	3 (25.0)	3 (17.6)	1 (100.0)
Number of surgeries performed based on clinical experience (*n* [%])				
1–5 years	0	15 (36.6)	19 (18.8)	0
6–15 years	0	15 (36.6)	64 (63.4)	0
16–39 years	56 (100.0)	11 (26.8)	18 (17.8)	22 (100.0)
Cervical dilation status (*n* [%])				
<6 cm	53 (94.6)	35 (85.4)	77 (76.2)	16 (72.7)
≥6 cm	3 (5.4)	6 (14.6)	24 (23.8)	6 (27.3)
Suture method (*n* [%])				
Barbed suture	22 (39.3)	19 (46.3)	55 (54.5)	14 (63.6)
Niche depth (mm)^a^	1.68 ± 1.31	1.91 ± 0.61	1.72 ± 1.06	1.96 ± 1.29
RMT (mm)^a^	7.94 ± 1.31	8.08 ± 1.94	8.84 ± 1.59	8.33 ± 2.36
Rate of niche (%)^a^	16.86 ± 10.98	19.32 ± 5.72	16.32 ± 9.67	19.36 ± 12.12
Conventional suture	34 (60.7)	22 (53.7)	46 (45.5)	8 (36.4)
Niche depth (mm) ^a^	2.98 ± 1.42	2.52 ± 1.01	2.58 ± 1.29	2.58 ± 2.02
RMT (mm)^a^	5.84 ± 1.74	9.27 ± 2.29	6.82 ± 1.72	7.63 ± 0.97
Rate of niche (%) ^a^	33.78 ± 14.39	21.68 ± 8.90	27.60 ± 12.22	23.18 ± 13.43

*Note*: Values are presented as mean ± SD or *n* (%) unless otherwise specified. Rate of niche calculated as (niche depth/[niche depth + RMT]) × 100.

Abbreviations: RMT, residual myometrial thickness; SD, standard deviation.

^a^ Mean ± SD.

## 4 DISCUSSION

Our sub‐analysis revealed multiple significant factors beyond suture type that influenced cesarean section outcomes. Specifically, RMT at 6 months post‐surgery was significantly affected by both the surgeons' years of experience and institutional factors. Notably, when comparing the effectiveness of barbed versus conventional suturing, significant inter‐institutional differences were observed (interaction test; *P*_interaction_ < 0.001). This finding underscores the importance of standardizing surgical protocols across institutions. Our results corroborate previous observations regarding regional and institutional variations in uterine incision closure methods and the impact of surgeon experience. The recently highlighted preventive effects of barbed sutures on CSD^12^, ^14^ may be related to the inter‐institutional differences revealed in our study, potentially reflecting varying degrees of surgical technique optimization across facilities. These results align with findings by Sholapurkar, who proposed an “ischemia and mal‐apposition hypothesis,” suggesting that surgical technique variations across institutions significantly impact cesarean scar healing outcomes more than patient‐specific factors.^25^ Similarly, Dominguez et al. emphasize the importance of surgeons' experience and training in the repair of cesarean scars, noting that individual variations in repair techniques often influence outcomes more than standardized facility protocols.^26^ Furthermore, the results support those of Tsuji et al., indicating that differences in practice between facilities (peritoneal closure approaches, suturing techniques, postoperative care protocols, etc.) contribute significantly to the incidence of cesarean scar defects across healthcare facilities.^5^ Although advanced cervical dilation and emergency cesarean sections during active labor are considered high‐risk factors for CSD,^27^ our data demonstrated that barbed sutures achieved superior outcomes even in these traditionally high‐risk cases. This finding represents a significant advancement in cesarean incision closure management, suggesting that barbed sutures may effectively mitigate conventional suture‐associated risk factors for uterine wall defects.

During the wound healing process, compromised blood flow and tissue weakness lead to chronic inflammation and subsequent scar formation.^28^ Our observed variations in outcomes related to surgeon experience and institutional factors likely stem from differences in suture tension control and appropriate spacing. Suboptimal execution of these technical elements may impair local blood flow and adversely affect wound healing.^29^

Recent surgical education in both western countries and Japan has seen reduced direct supervision opportunities due to advances in surgical materials and work‐hour restrictions based on labor standards.^30^ Consequently, simulation‐based education has gained increasing importance.^31^ Our findings support the value of simulation training in cesarean section procedures, with the experience‐dependent outcomes aligning with previously reported case number requirements.^10^

Our findings regarding barbed suture usage have important implications for improvement in the quality of surgical education. This suggests that the use of barbed sutures may mitigate technical disparities and shorten the learning curve. However, our findings regarding the persistent experience‐related differences in surgical outcomes, sustained inter‐institutional variations, and observed interaction effects emphasize the continued importance of standardized training protocols and ongoing surgical education. Skill acquisition demands comprehensive education, and sustained practice is vital for achieving expertise in specialized fields. Abdullatif et al. performed a randomized clinical trial evaluating simulator‐based instruction in urological procedures across 10 nations and showed accelerated competency development and decreased complication rates in the simulator‐trained cohort.^32^ Advanced simulation systems, like those created by Uemura et al., evaluate suturing technique quality and facilitate rapid skill enhancement.^33^ For cesarean delivery operations, guidelines suggest completing 10–40 procedures under expert supervision before transitioning to independent practice.^10^ Consequently, uterine training models permitting repeated practice without patient involvement likely offer significant educational value.

Our study has several key limitations that warrant consideration in future research. First, the limited number of participating centers (*n* = 4) restricted our capacity to fully examine how institutional factors influence surgical outcomes. And a significant limitation was our lack of standardized criteria for classifying the four participating centers beyond basic care level designations. Without systematic documentation of facility‐specific variables—such as surgical volume, equipment differences, and team composition—we cannot precisely identify which institutional characteristics influenced surgical outcomes the most. This constrains interpretation of the “facility factors” that our analysis found significant for residual myometrial thickness results.

Second, recruitment was unevenly distributed, with institution C accounting for nearly half (46%) of all participants. This imbalance likely introduced sampling bias and may have oriented our findings toward the specific practices and outcomes of this dominant institution, potentially reducing broader applicability. This represents a classic selection bias, specifically recruitment bias, where participant distribution across study sites lacked proportionality. Additionally, the inability to blind surgeons to the suture type represents an important source of selection and performance bias. Our results may also exhibit cluster effects, where outcomes within individual institutions show greater similarity to each other than to those at other sites, a recognized challenge in multicenter studies with few participating locations. Future studies should establish comprehensive facility classification criteria to better elucidate specific institutional determinants of cesarean closure outcomes.

## 5 CONCLUSION

Institutional factors and surgeon experience represent significant determinants of residual myometrial thickness when using barbed sutures for cesarean closure, highlighting the importance of standardized surgical protocols and training across facilities. Further studies are warranted to elucidate the specific factors that most strongly influence these outcomes.

## AUTHOR CONTRIBUTIONS

Conceptualization: JM. Writing—original draft preparation: JM. Methodology: JM, HO. Data collection: JM, TM, HN, AS, CK, SS, SM, AO, EE. Review and editing: JM, TM, HO, HM. All authors read and approved the final manuscript.

## FUNDING INFORMATION

This work was supported by the internal clinical research incentive funds (grant numbers CRB19‐006 and 206002). These were internal grants from our institution. This study did not receive external funding from any other agency.

## CONFLICT OF INTEREST STATEMENT

JM was a member of the Speaker's Bureau for Ethicon, Johnson & Johnson, New Brunswick, NJ, USA in 2022–2024. The remaining authors have no conflicts of interest.

## INFORMED CONSENT

Our manuscript contains participant data. Consent for publication was obtained from each individual.

## Supporting information


Data S1

Data S2

Data S3

Data S4

Data S5

Table S1:

## Data Availability

The datasets generated and/or analyzed during the current study are not publicly available but will be available from the corresponding author upon reasonable request.
