# Supplementary material for: Determinants of residual myometrial thickness after cesarean delivery: Comparative analysis of barbed versus conventional sutures—A sub‐analysis from the SPIRAL trial
Source: Int J Gynaecol Obstet. 2025 Jun 5;171(2):861–8. doi: 10.1002/ijgo.70273 (PMC12553111; doi:10.1002/ijgo.70273)
Supplement: Supplementary file 1 — Data S1 [file IJGO-171-861-s006.pdf]

Contemporary Clinical Trials 107 (2021) 106449

Contents lists available at ScienceDirect

Contemporary Clinical Trials

journal homepage: www.elsevier.com/locate/conclintrial

The Spiral Trial: A multicenter, randomized, controlled trial of Spiral thread sutures versus conventional thread sutures to prevent thinning of uterine scars following elective cesarean section

Jota Maki [a,*], Hikari Nakatou [a], Kazumasa Tani [a], Eriko Eto [a], Kei Hayata [a], Dan Yamamoto [b], Kenji Kai [b], Takashi Tamada [c], Kazuyo Akamatsu [c], Kunihiro Kawanishi [d], Keiichiro Nakamura [a], Hisashi Masuyama [a]

[a] *Department of Obstetrics and Gynecology, Okayama University Graduate School of Medicine, Dentistry and Pharmaceutical Sciences, 2-5-1 Shikata-cho, Kita-ku, Okayama 700-8558, Japan*
[b] *Department of Obstetrics and Gynecology, National Hospital Organization Fukuyama Medical Center, 4-14-17, Okino-kamicho, Fukuyama City, Hiroshima 720-8520, Japan*
[c] *Department of Obstetrics and Gynecology, Iguchi Perinatal and Obstetrics and Gynecology Hospital, 71-4 Kambe-cho, Fukuyama City, Hiroshima 720-2122, Japan*
[d] *Department of Obstetrics and Gynecology, Yashima General Hospital, 2105-17 Yashima-Nishi-machi, Takamatsu City, Kagawa 761-0113, Japan*

ARTICLE INFO

Keywords:
Suture techniques
Cesarean section
Scars
Pregnancy
Transvaginal ultrasound
Myometrium

ABSTRACT

Background: The aim of this randomized controlled trial (RCT) is to investigate whether Spiral-thread sutures are superior to conventional sutures (0-Vicryl) for preventing uterine scar thinning following elective cesarean section.

Methods: This multicenter, parallel-group RCT will be conducted in four hospitals across three medical regions in Japan to assess 200 women (≥20 years old) with singleton pregnancies who are scheduled to undergo cesarean sections. Eligible women will be randomly assigned (1:1 ratio) to receive either the conventional uterine suture continuous absorption thread, which is most commonly used in Japan, or the Spiral thread. The primary endpoint is the degree of scar thinning, measured by transvaginal ultrasonography 6–7 months postoperatively, to evaluate the position of the uterus (anterior or posterior tilt) and myometrial wound thickness. The degree of thinning will be compared between the groups, and four measurements (mm) of the thinning area, including caudal distance, depth of the depression, remaining thickness of the myometrium on the serous side of the most depressed area, and width of the depression, will be recorded in the sagittal view on transvaginal ultrasound. Secondary endpoints will include total operative time, suture application time (from birth to the end of uterine suturing), operative blood loss, number of additional *Z*-sutures or continuous sutures required to stop bleeding, maternal abnormality frequency (surgical complications and postoperative infections), surgeon's years of experience, and clinical interpretation of individual subscale scores.

Discussion: This study shall provide important evidence on the optimal suture for preventing hysterotomy wound thinning after the first cesarean section.

Trial registration: National Institute of Public Health, Japan: jRCT1062200001 (May 7, 2020; https://rctportal.niph.go.jp/en/detail?trial_id=jRCT1062200001) and Okayama University Certified Review Board: CRB6180001 (April 9, 2020, version 3.0).

List of abbreviations

0-Vicryl	conventional uterine suture-absorbing thread
C-section	cesarean section
jRCT	clinical research protocol and research summary disclosure system
RCT	randomized controlled trial

1. Introduction

Cesarean sections (C-sections) are very common obstetric and

* Corresponding author.
E-mail address: phrg4irh@okayama-u.ac.jp (J. Maki).

https://doi.org/10.1016/j.cct.2021.106449
Received 4 February 2021; Received in revised form 17 April 2021; Accepted 17 May 2021
Available online 20 May 2021
1551-7144/© 2021 Published by Elsevier Inc.

gynecological procedures, and the number of operations is on the rise worldwide. Currently, the C-section rate in Japan exceeds 25% [1], and as many as 250,000 women a year undergo a C-section for a variety of reasons. In some countries, 50% of all births are performed via C-section [2]. In Japan, about 7%–12% of women exhibit obvious muscle-layer thinning in the C-section scar area, which may cause infertility and pose a high risk for the next pregnancy, hypermenorrhea, dysmenorrhea, and other factors that affect the quality of life [3–6]. This concept has recently been termed C-section scar syndrome and calls for attention. Different C-section sutures are being developed in the attempt to prevent C-section scar syndrome, as well as treatment methods for severe cases. In other countries, single-ligation sutures, which tend to prolong the operative time and increase the use of medical resources, are not recommended [7–11]. In past literature, there was no statistically significant difference in the prevention of thinning between single ligation and two consecutive layers of sutures. Currently, there is no standard policy to prevent muscle-layer thinning worldwide.

In November 2017, an antimicrobial, monofilament, absorbable suture with unidirectional barbs on the surface called "Spiral thread" was launched in Japan. This suture is used in laparoscopic myomectomy in Japan and was suggested to have an excellent hemostatic effect on the myometrium and prevent suture failure [12–14]. Although several trials have been conducted to investigate the effectiveness of Spiral threads in reducing operative time and blood loss [15–17], there have been limited trials in which they were compared to conventional absorbable threads (0- Vicryl) [18], which is the worldwide standard for uterine wound closure.

The purpose of this randomized controlled trial (RCT) is to investigate whether uterine scar thinning can be more effectively prevented using Spiral threads than using conventional threads. We also aim to identify suturing methods that are effective in preventing thinning of the uterine scar area and eliminating collateral problems.

2. Patients and methods

2.1. Study design and setting

In this study protocol (version 3.0, May 7, 2020), we describe the design of a multicenter, parallel-group RCT (1:1 ratio) planned to be conducted at four obstetrics and gynecology departments across all three healthcare regions in Japan. To ensure transparency and accountability in clinical research, it is necessary to have a quality protocol in place before the beginning of the study. A quality protocol facilitates proper conduct with, reporting of, and external review of clinical research. The study protocol conforms to the SPIRIT guidelines [19] and the RCT will conform to the CONSORT 2010 checklist for reporting randomized trials [20]. This study protocol was prepared according to the SPIRIT checklist (Additional File 1). The obstetrics departments at Okayama University Hospital, National Hospital Organization Fukuyama Medical Center (Hiroshima), Iguchi Perinatal and Obstetrics and Gynecology Hospital (Hiroshima), and Yashima General Hospital (Kagawa) will recruit pregnant women for the study. The study was registered at the Japan Registry of Clinical Trials (jRCT1062200001) on May 7, 2020, and was approved by the Okayama University Certified Review Board (CRB19–006) on April 9, 2020. Enrollment started at the first hospital in May 2020 and at the last hospital in June 2020, and recruitment is expected to finish in September 2023 (research participant registration deadline: March 31, 2022).

2.2. Patients

Two hundred pregnant women aged 20 years or above, referred to the obstetrics department at one of the four hospitals mentioned above, and who fulfill the eligibility criteria below will be included.

Oral and written information about the study will be provided by the examining doctor. The examining doctor will provide this information to primiparous or multiparous women without a history of C-section.

2.3. Eligibility criteria

2.3.1. Inclusion criteria

Women who meet all the following criteria will be included:

(1) Age 20 years or older when providing consent.
(2) Written, informed consent provided for participation after receiving a thorough explanation of the study.
(3) Pregnant women in whom a C-section is being performed for the first time, and who are scheduled to undergo an elective C-section after being hospitalized in the obstetrics and gynecology departments of the hospitals in which the study is conducted. C-sections are performed after 36 weeks of gestation due to the following reasons: a breech (pelvic position), problematic fetal and/or maternal conditions, or prolonged delivery, and an initial C-section is defined as a C-section performed for the first time.
(4) Approval provided for the researcher to obtain information on the infant from their medical records for research purposes.

2.3.2. Exclusion criteria

Women who meet even one of the following criteria will be excluded:

(1) Relocation to another hospital for delivery.
(2) Any of the following complications during pregnancy:
   - o Blood disorders or pregnancy with coagulopathy
   - o Multiple pregnancies
   - o Mental illness
   - o Incision other than lower uterine transection
   - o Placenta previa
   - o Postpartum hemorrhage (bleeding more than 2000 mL)
   - o Emergency C-section after the full opening of the uterine ostium
   - o Adenomyosis of the anterior wall
   - o Myoma within the incision
(3) Hypersensitivity to constituent metals such as stainless steel, chromium, or nickel.
(4) Inappropriate for the study (poor understanding/objection to the study procedures) in the investigator's opinion.

2.4. Recruitment procedure

The overall trial flow is outlined in Fig. 1. Women referred to the obstetrics department at one of the four study hospitals and complying with the eligibility criteria will be invited to participate in this RCT study. The doctor in the study group involved in recruitment has been trained and instructed on the recruitment procedure. The study information is also available in the form of a QR code for the participants to study at home or at the hospital, before deciding whether to participate in the study.

The cost of the Spiral and Vicryl suture threads used in this study will be covered by normal medical insurance. The price of the Spiral and Vicryl suture threads per unit is generally determined based on the principle of supply and demand. Spiral is an open price, and Vicryl is a regular retail price of 8.25 USD per unit. There will be no additional cost to the study participants for their participation. Although the threads to be used in the two groups are different, there is no difference in the cost of insurance treatment. Transvaginal ultrasonography is included in the 1-month postpartum examination, while postoperative follow-up at 3–4 months and 6–7 months is covered by insurance, as women are at an increased risk of disease during this period, and follow-up is necessary. As transvaginal ultrasonography will be mandatory for participants, this will be conveyed to potential participants when seeking their informed consent. In addition, all participants will be assessed for uterine thinning

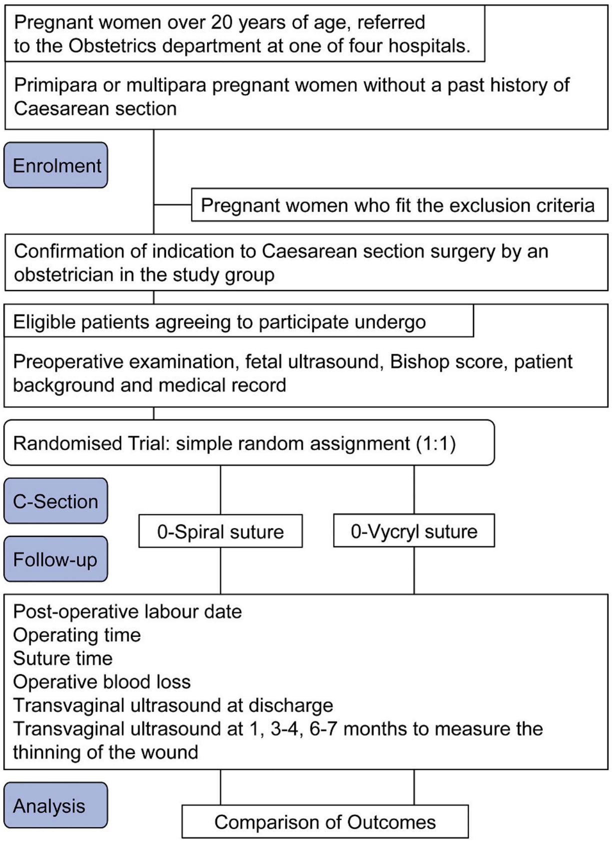

Fig. 1. Patient flow through the study. RCT, randomized controlled trial.

after the C-section, abnormal findings of postoperative medical examinations will be identified, and the risk to future pregnancies and participants' life will be indicated to them. There is no honorarium for participation in this study.

2.5. *Intervention period per participant*

The expected period of study participation is from the time of obtaining consent to the 6–7-month check-up. The intervention period will be from randomization to the performance of the C-section.

2.6. *Randomization procedure*

Each participant will be enrolled by the principal investigator or sub-investigator (hereinafter referred to only as "investigator") and given a participant identification code. At the time of surgery, either a Spiral thread or a Vicryl thread will be used, as assigned via simple randomization. The investigator will enroll and allocate cases via the following steps:

(1) Obtain written consent.
(2) Fill out the list of participant identification codes, including the date of obtaining consent and any other information necessary to match the participant with the corresponding identification code. The codes will be assigned in numerical order of case enrollment.
(3) Assign the participant to a treatment group using the centralized registration system installed at the Department of Obstetrics and Gynecology, Okayama University. The allocator will issue an identification code for each participant (different to that assigned by the investigator) in the order of enrollment and will use the randMS® mobile application (sold by Shinji Maeda, created using Filemaker® and Filemaker pro advanced®, ©2019 kazenoan) [21].
(4) Inform the person in charge of case enrollment at each hospital of the new enrollment by e-mail from the headquarters.

2.7. *Blinding*

Participants will be blinded to the intervention they will receive; however, as the treatment providers cannot be blinded, this will be a single-blinded study.

At each institution, a research collaborator will be assigned to manage the threads that will be used for the sutures. The physician in charge will inform collaborators of the participant's allocation and the collaborators will provide the type of thread allocated.

The investigators at each site will immediately notify the site principal investigator of any participant who withdraws consent or whose participation is discontinued.

2.8. *Interventions*

Pregnant women fulfilling all eligibility criteria will be randomly assigned to one of two groups in a 1:1 ratio. One group will receive the conventional uterine suture continuous absorption thread (0-Vicryl), which is the type most commonly used in Japan; the other group will receive the Spiral thread.

2.9. *Treatment*

2.9.1. *C-section surgery*

A transverse incision, approximately 10 cm in length, will be made in the lower part of the uterus. Following delivery, two pieces of either 0-Spiral PDS plus or 0-Vicryl thread will be used for uterine repair. The principal investigator will visit each institution to demonstrate the technique in advance, to ensure consistency in the surgical procedure across institutions. A procedure manual has been prepared and will be given to each medical institution to the same end. In the manual, physicians are instructed that both ends of the wound should be single-ligated, followed by the application of a continuous, two-layer suture (Fig. 2). Physicians are also instructed that these sutures should not be used for longer than 6 weeks in tissues that require a prolonged period of fusion, such as the fascia [22,23]; this information is not applicable to the uterus. A list of standardized sutures is provided in Fig. 2.

2.9.2. *Medical equipment used for the intervention*

0-STRATAFIX® Spiral PDS plus (0.40–0.499-mm polydioxanone sutures; Ethicon division of Johnson & Johnson, New Brunswick, New Jersey, U.S.), Japanese Medical Device Nomenclature [JMDN] code: 16584000, approval number: 22900BZX00123000) [22] or 0-Vicryl (Ethicon division of Johnson & Johnson), (0.35–0.399-mm polyglactin sutures; JMDN code: 17471000, approval number: 15700BZY01341000) [23] will be used. The 0-Spiral thread is one size thicker than the 0-Vicryl thread of the same size for the inclusion of the barbs. Two layers of sutures (two sutures) will be used, each packaged for one dose (one thread) and stored at room temperature.

The management protocol for the Spiral and Vicryl thread used in this study has already been approved by jRCT, and appropriate storage and other controls will be performed in accordance with the requirements for approval of medical devices.

2.10. *Follow-up*

2.10.1. *Participants' background*

For participants, the following variables will be collected: age, history of the present illness, complications, medical history, the reason for C-section, number of weeks of pregnancy at the time of surgery, uterine cavity opening (cm), degree of uterine retraction (%), weight, body mass

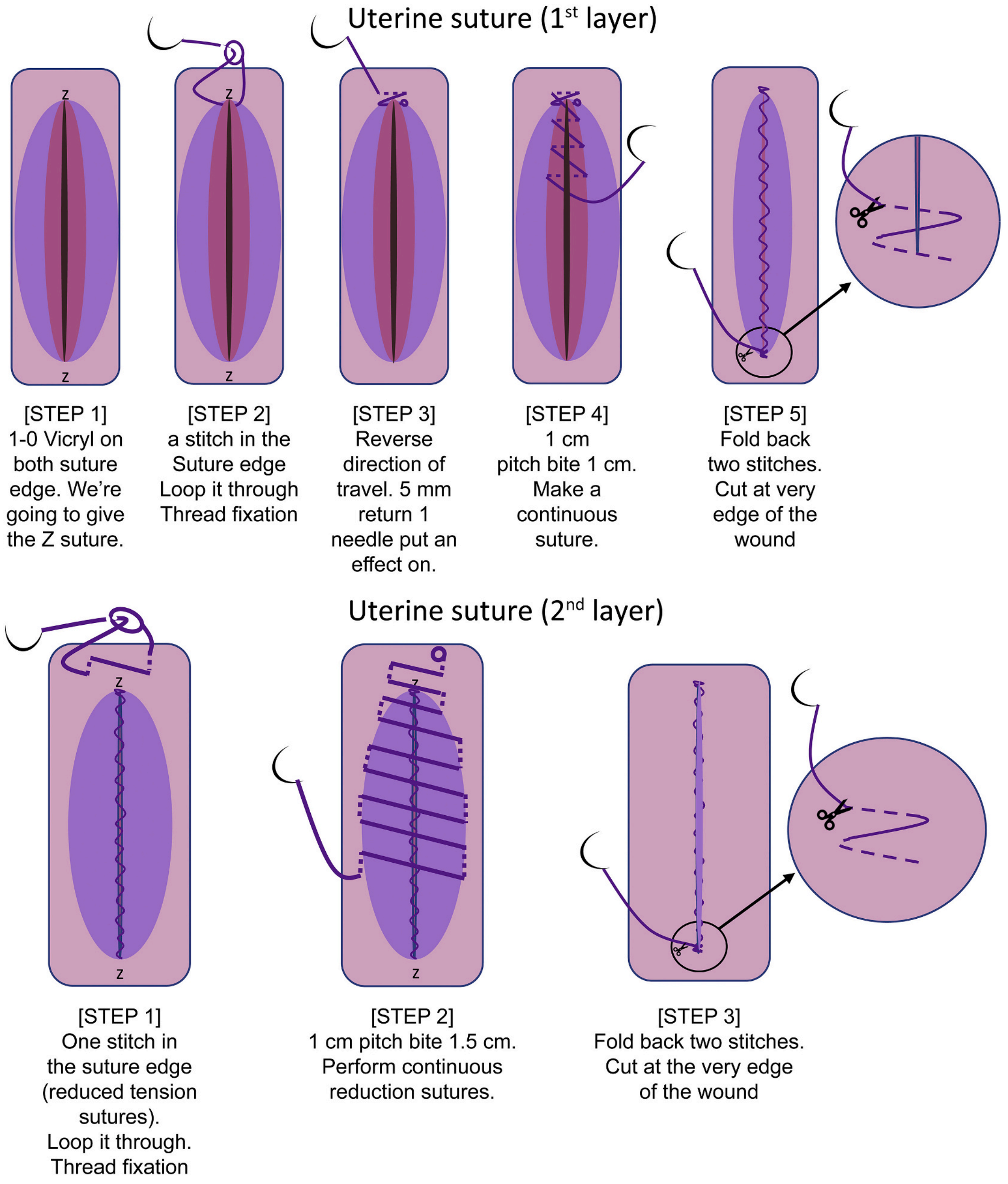

Fig. 2. A procedure manual has been prepared and will be given to each medical institution to ensure consistency in technique performance in both groups across institutions.

index, weight gain, treatment, and perinatal course.

For the delivered children, the following variables will be collected: transabdominal ultrasound fetal-measurement data (estimated weight, fetal position, and orientation), sex, birth weight, height, umbilical arterial blood gas analysis (pH), presence of a neonatal abnormality, and Apgar score.

The size of the hysterotomy site increases with the size of the baby, and boys are typically larger than girls. The size of the fetus is a risk factor for blood loss during surgery [18], and thus, it is necessary to collect data immediately after birth.

2.10.2. *Transvaginal ultrasound*

The timetable of transvaginal ultrasounds is included in Table 1. Transvaginal probes with a center frequency of 7.5 MHz will be used for all transvaginal ultrasound examinations. All ultrasounds will be performed by physicians who received uniform training and certification prior to patient enrollment. Practice guidelines for the measurement and subjective assessment of all four uterine wounds using transvaginal ultrasound at 6–7 months (i.e., the primary endpoint) will be given to each physician, who will also be asked to carefully read the detailed description of the study. The principal investigator will standardize the ultrasound settings at each institution and provide direct supervision at each institution at least once. Each transvaginal ultrasound will be performed according to a predefined protocol [19]. The patient will be placed in the lithotomy position. A vaginal probe covered with a sterile lubricating condom will be inserted and advanced along the vaginal canal until an appropriate sagittal section of the uterine wound, from the cervix to the corpus, is visible. The probe will be rotated 90 degrees to the left from the position in which the sagittal section is depicted. The probe will then be slowly withdrawn in that position until the best image is obtained. The size of the image should be consistent between patients. The wound will be measured three times and findings documented. The study physician will evaluate the quality of the three images and record their subjective decision of which measurement is the most accurate. If

Table 1
Overview of data collection.

Dates	First visit to target facility	Preoperative blood collection	On admission	Cesarean section - pre-discharge	1-month check-up	3–4-month check-up	6–7-month check-up
Tolerance	–	−30 days to the 0 days	+3 days	+3 days	−10 days to +10 days	−15 days to +15 days	−15 days to +15 days
Eligibility confirmation[a]	●		●				
Consent acquisition	●						
Registration	●						
Use of research medical devices				●			
Background on pregnancy		●	●	●			
Background on infants				●			
Transvaginal ultrasonography[b]				●	●	●	●
General maternal blood test		●		●			
Intraoperative evaluation				●			
Confirmation of subjective symptoms				●	●	●	●
Identification of adverse events and side effects[c]				●	●	●	●

Each item labeled as a dot in the table will be obtained after obtaining consent.

[a] Eligibility verification means that patients are verified to be medically and socially fit to enter the study, considering their age, pre-existing medical conditions, and comorbidities.

[b] Transvaginal ultrasonography will be used to observe the uterus after the cesarean section is completed, at 6 days (at discharge) and at the 1-month, 3–4-month, and 6–7-month post-discharge check-ups. These will be performed to confirm the safety and effectiveness of the intervention.

[c] Adverse events are all undesirable events, such as side effects, regardless of their causal relationship to sutures.

the image quality is similar among measurements, the image in which the neck length was shortest will be used. Where thinning in the depressed area recorded at 6–7 months is less than 1 mm, it will be recorded as 1 mm.

2.10.3. Subjective symptoms

The presence or absence of dysmenorrhea and chronic pelvic pain will be confirmed by interviewing the participants during the follow-up visits.

2.10.4. Adverse events and side effects

For each adverse event, the following will be described in a case report form for each participant: its nature, time of onset and resolution, extent, treatment, outcome, assessment of severity, and relevance of the Spiral or Vicryl threads used for uterine suturing. If necessary, a follow-up examination will be performed. However, thinning of the uterine scar is a side effect that always occurs after a C-section, and the main objective of this study is to investigate the extent of this side effect.

2.11. Crossover and discontinuation

An investigator may discontinue the research on an individual participant for any of the reasons described below. The reason for discontinuation will be explained to the participant, as necessary. In addition, research participants will be treated in good faith after discontinuation.

The nature of the adverse event will be determined based on the accompanying documentation and interview form [24,25]. If the nature, severity, or frequency of the adverse event does not fit that of one of eight adverse events defined below, it will be considered an unknown adverse event. Defined adverse events will include: 1) allergic reactions due to hypersensitivity to stainless steel or constituent metals, such as chromium, nickel, or triclosan; 2) suture failure in those with slow wound healing due to poor nutrition, weakness, anemia, uncontrolled diabetes, or infection; 3) allergic reactions in those with a history of such reactions, including those who have been diagnosed with chronic allergies; 4) a minor, acute inflammatory reaction, hemorrhage, tissue reaction, granulation, or fluid accumulation at the suture sites; 5) formation of keloids at the suture sites; 6) suture protrusion and delayed absorption possibly associated with poor blood supply; 7) wound infection; and 8) foreign body reactions. The incidence rate of foreign body reactions is unknown.

Adverse events will be graded from 1 to 5 according to the Common Terminology Criteria for Adverse Events [26].

2.12. Criteria for discontinuation of research

(1) When a research participant declines to participate in the research or withdraws his or her consent.
(2) If the entire study is canceled.
(3) When the physician in charge of the research decides that it is appropriate to discontinue the research for other reasons.

2.13. Data collection

A timetable of data collection is provided in Table 1. Data will be collected within 30 days preoperatively, on admission, at the time of surgery, and at outpatient visits at 1, 3–4, and 6–7 months after discharge. Data collection and recording will be performed by the principal investigator or by a subcontractor. The obtained data and a list of participant identification codes will be sent to the secretariat by e-mail once every three months, protected with a password. All data collected will be entered into Excel 2019 and protected with a password (Microsoft Corporation, Redmond, Washington, USA).

2.14. Outcomes

2.14.1. Baseline characteristics

Baseline characteristics to be collected are described in the participants' background (see previous section "Participants' background").

2.14.2. Primary outcome

The degree of thinning of the scar will be evaluated and compared between the groups. It will be measured using transvaginal ultrasonography at 6–7 months after the operation, to analyze the position of the uterus (anterior or posterior tilt) and the thickness of the myometrial wound, as follows. The degree of thinning will be assessed by obtaining four measurements (mm) on a transvaginal ultrasound sagittal section: ① caudal distance, ② depression depth, ③ thickness of the remaining myometrium from the most depressed area to the plasma membrane, and ④ width of the depression in a transverse section. The thickness of the remaining myometrium (③) will be calculated as a percentage of the total myometrial thickness (② + ③). A comparison of ① to ④ will be made between the two groups (Fig. 3).

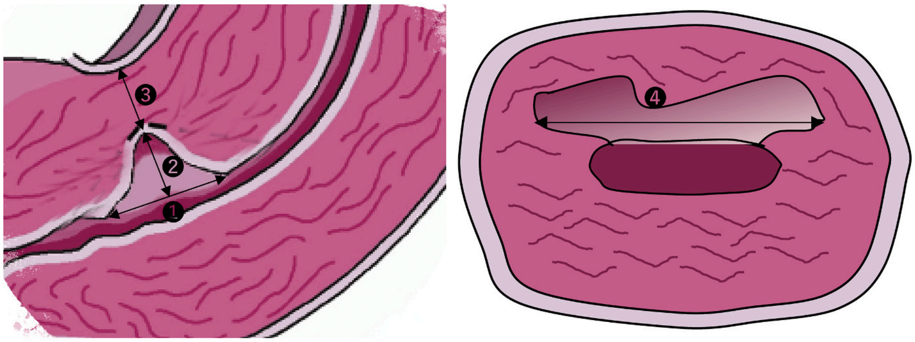

Fig. 3. The degree of thinning will be assessed by obtaining four measurements (mm) on a transvaginal ultrasound sagittal section.

2.14.3. Secondary outcomes

Secondary outcomes include operative time from the start of surgery to the end of abdominal closure, the duration from delivery to completion of uterine closure, blood loss during surgery from the start of surgery to the end of abdominal closure, the number of *Z*-sutures used to stop bleeding in addition to the continuous sutures, the frequency of maternal abnormalities (complications at the time of surgery, presence or absence of postoperative infection), and safety (presence or absence of serious adverse events).

2.14.4. Other outcome measures

The incidence of abnormalities during the treatment and the perinatal course of the abnormalities will also be analyzed.

2.14.4.1. Hematoma occurrence. The occurrence of a hematoma on the anterior surface of the uterine wound on transvaginal ultrasonography at the time of discharge from the hospital and at the 1- and 3-month postoperative examinations will be documented.

2.14.4.2. Postoperative fever incidence. For participants with a postoperative body temperature of 38 °C or higher, the presence of fever will be considered. In cases where the cause of fever is suspected to be an intra-abdominal infection, broad-spectrum antibiotics will be prescribed.

2.14.5. Presence or absence of dyspraxia and chronic pelvic pain

The rate of menstruation at 6–7 months and the presence or absence of dyspraxia and chronic pelvic pain will be confirmed by interviewing the participants.

2.15. Data management

The principal investigator will record the following:

(1) Identification code of the study participants
(2) Matters related to medical treatment and participants' examination
(3) Matters related to participation in this study
(4) Other matters necessary to conduct this research.

Information collected in this study, essential documents pertaining to the conduct of the research (research protocol, documents pertaining to the explanation to and consent of the study participants, summary report, documents or copies of documents prepared by the principal investigator, documents pertaining to the review and opinion received from the Accredited Clinical Research Review Board, documents pertaining to monitoring, documents pertaining to the research, and documents related to the research, etc.), as well as a list of participant identification codes, consent forms, case report forms, and other documents or records necessary to assure the reliability of the data (which will be used in this study) will be stored in an access-controlled location for 5 years after the end of the study, and then shredded and disposed of with great care to protect personal information. Any information stored on computers will be deleted completely.

2.16. Data monitoring

Monitoring of the study will be carried out by a monitor designated by the principal investigator, in accordance with the standard operating procedures for monitoring at medical institutions. The monitoring target will be 5/200 cases. However, the sampling rate will be increased to 5% of the total number of cases if at least one participant develops complications. Monitoring will include verifying the progress of the clinical trial and confirming whether all required documents related to the procedure are present at the research office by contacting the office electronically. The monitor's reports will be made available to the Okayama University Certified Review Board, as necessary. It will describe the items to be complied with during preparation, inspection, and submission. If deviations or data errors are found during monitoring, the monitor will inform the principal investigator of any doubts and urge them to take appropriate action, such as confirming the data or requesting revisions to the research protocol.

2.17. Adjudication committee

The principal investigator will report the following information on the progress of the study to the Okayama University Certified Review Board every year from the date of submission of the research plan to the Minister of Health, Labour and Welfare, and within 2 months after the expiration of the research period:

(1) The number of participants participating in the study
(2) Occurrence and subsequent course of adverse events related to this research
(3) Occurrence of nonconformity with the ministerial ordinance or research plan pertaining to this research, and details of subsequent actions
(4) Evaluation of the safety and scientific validity of this study
(5) Involvement of the manufacturer or distributor in this research

2.18. Sample size calculation

The total number of participants in all study centers is planned to be 100 in the Spiral group and 100 in the Vicryl group, for a total target of

200 participants at the four study institutions.

Two different methods were attempted to examine the number of cases. Firstly, epidemiological studies have shown that the prevalence of scar thinning after C-sections is about 7% [27]. In previous studies [18,28,29], intraoperative blood loss using Spiral versus conventional sutures was examined using approximately 100 cases, randomly allocated to each group in a 1:1 ratio. Zayed et al. [18] conducted a pilot study for sample size calculations, in which they observed the difference in uterine closure time between the two groups. Their calculation indicated that each group required a sample of 48 patients to detect such a difference with an alpha error of 5% and a beta error of 20%. They expected a dropout incidence of about 5%; thus, 50 patients were included in each group [18].

Secondly, in a previous study in which Vicryl threads were used [29], the mean thickness of the C-section scar, the primary endpoint, was 4.18 mm, with a standard deviation of 1.76 mm. Based on our experience with Spiral threads, we expect that the thickness of the C-section will be at least 1 mm larger than that with conventional threads. We also expect that the standard deviation of the thickness of the C-section scar will be greater in our multicenter study than that in previous studies conducted in single centers. Assuming that the mean thickness of the C-section scar with Vicryl and Spiral threads is 4.18 mm $\pm$ 2.4 mm and 5.18 mm $\pm$ 2.4 mm, respectively, we calculated the number of participants required for $\alpha = 0.05$ and $\beta = 0.20$. According to the sample size calculation using Stata Statistical Software (Release 16; StataCorp LLC, College Station, Texas, USA), 92 subjects were required per group (184 in total). Based on the above, we considered the two types of methods and chose the latter, which has more cases. To account for a dropout rate of about 5%, we set the required number of subjects at 100 per group (200 in total).

2.19. Statistical analysis

Cross-sectional data for all patients will be recorded over six months. The target population for analysis will be the "Full Analysis Set," defined as all participants who start the study treatment. Cases for which there are missing data for any of the ultrasound examinations will not be used for analysis; enrollment will continue until there are 100 cases in each group for which we have complete 6–7-month data. Data that cannot be measured or are clearly outliers will be excluded for analysis. All patients enrolled in the study will be included in the secondary evaluation analysis, even if their ultrasonography results are not available. The analysis will be performed at the Department of Obstetrics and Gynecology, Graduate School of Medicine, Dentistry and Pharmaceutical Sciences, Okayama University. The validity of the analysis will be verified by the Center for Innovative Clinical Medicine and the Department of Epidemiology of Okayama University, as a third-party institution.

Analyses will be performed using the Chi-Square test, the *t*-test, or Mann-Whitney *U* test to use a test that matches the content of the survey item, for comparison of patient baseline results, ultrasound results, presence or absence of adverse events, presence or absence of ultrasound abnormalities, and the main and secondary endpoints in the Spiral and Vicryl groups. Scar size will be presented as quartiles, and the size of each C-section scar (i.e., the primary efficacy endpoint) will be compared between the two groups to estimate the involvement of thread type in preventing thinning.

2.20. Ethics, dissemination, and perspectives of the study

All researchers involved in this research shall conform to the provisions of the Declaration of Helsinki, as revised in Fortaleza, Brazil, in October 2013, as well as to the Japanese Clinical Research Act and related regulations. Informed consent will be obtained from all patients. When handling information related to the conduct of research, the researcher will attach to it a research participant identification code unique to the study, giving due consideration to protecting participants' confidentiality. When the results of the research are made public, participants' names, dates of birth, and other information that can be used to directly identify them shall not be included. In addition, participant information obtained in the study shall not be used for purposes other than those of research.

2.21. Conflicts of interest related to research during the research period and individual earnings

In accordance with the "Guidance for Management of Conflict of Interest under the Clinical Research Act," [30] the principal investigator shall declare any conflicts of interest and obtain review and approval for them. In addition, any changes in conflicts of interest of the research group or of individual investigators will be reviewed when periodic reports are made to the Okayama University Certified Review Board.

2.22. Dissemination and protocol amendments

2.22.1. Method of disclosure of information on research (registration of research plan and publication of research results)

This study's results will be registered in the clinical research protocol and research summary disclosure system (jRCT). The results of this study will be presented at the Japan Society of Obstetrics and Gynecology and published as an article in a professional journal, such as *Obstetrics & Gynecology*. Published results will be limited to those that undergo statistical processing.

2.22.2. Protocol amendments

Information necessary for the safe conduct of clinical research will be collected and reviewed. If new safety guidelines become available, the research protocol and consent documents will be revised as necessary. Any changes or revisions to the research protocol or consent documents will be approved in advance by the Accredited Clinical Research Review Board. When the following minor changes are made to the implementation plan, the details of the changes will be notified to the Okayama University Certified Review Board within 10 days of the changes:

(1) Notification of changes to the implementation plan
(2) Revision of the implementation plan
(3) Notification of review results by the Okayama University Certified Review Board

2.23. Perspectives of the study

The results of this RCT will provide high-quality evidence supporting or opposing the hypothesis that the use of Spiral threads reduces scar thinning compared to traditional threads. Our results may also help identify new sutures to prevent thinning of the uterine scar area after C-section.

3. Discussion

In this prospective, multicenter clinical study, we will compare the degree of uterine scar thinning between participants whose C-section wounds are closed using Spiral threads and those receiving conventional sutures. We believe that our results will contribute to the prevention of medically induced thinning of uterine scars.

The strengths of this study are that firstly it will provide evidence concerning the optimal suture type for preventing post-hysterotomy wound thinning after a woman's first C-section. Secondly, this multicenter study will include different kinds of facilities in which deliveries are performed, ranging from primary to higher-care facilities, and viable and clinically appropriate suture techniques will be compared. Thirdly, the suturing method we will use, the two-layered continuous suture, is the most commonly used suturing method internationally; it yields a

better short-term prognosis and may reduce surgical wound thinning at a general level. Moreover, the only difference between the use of Spiral thread and the usual two-layer continuous suture is that it does not require any ligation. There is no need for the surgeon to learn a new suturing technique; we can continue to use the same continuous sutures as before. If the Spiral thread proves to be able to prevent thinning of the uterine wound in first-time cesarean patients, it has the potential to spread worldwide.

On the other hand, the limitation of this study is that this is a single-blinded study; considering the type of intervention, the treatment providers cannot be blinded.

Ethics approval and consent to participate

This study has been approved by the Regional Committees of the Japan Registry of Clinical Trials, the National Institute of Public Health, and the Okayama University Certified Review Board (CRB19-006). Patients will provide written informed consent at the time of enrollment.

Consent for publication

Our manuscript may contain any participants' data in any form (including any details, images, or videos); consent for publication must be obtained from that person.

Availability of data and materials

The datasets generated and/or analyzed during the current study will not be publicly available but will be available from the corresponding author upon reasonable request.

Funding

This work will be supported by Funding incentive funds [grant number; CRB19-006;206002]. It will not receive any external funding from other agencies.

Authors' contributions

All authors read and approved the final manuscript.

Declaration of Competing Interest

The investigators have no competing financial interests or personal relationships that could influence their conduct in this study.

Acknowledgements

We thank the obstetrics surgeons and other healthcare personnel involved in the recruitment, testing, and treatment of patients at the Department of Obstetrics and Gynecology, Okayama University Hospital; The Department of Obstetrics and Gynecology, National Hospital Organization Fukuyama Medical Center; The Department of Obstetrics and Gynecology, Iguchi Perinatal and Obstetrics and Gynecology Hospital; and The Department of Obstetrics and Gynecology, Yashima General Hospital. We also thank the staff in all the hospitals' operating theaters and perinatal wards. We thank Associate Professor Jun Sakurai and Assistant Professor Toshiharu Mitsuhashi, at the Center for Innovative Clinical Medicine and the Department of Epidemiology in Okayama University, for their statistical support during study planning, and for promising such support for the final data analysis. The central study coordinator is Assistant Professor Shihoko Nanba. The study advisory board with whom we consulted during study planning, and with whom we will consult during result interpretation and other relevant matters are as follows: Professor Katsuyuki Hotta, the Okayama University Certified Review Board; Professor Yoshinobu Maeda and Associate Professor Naruto Taira, Okayama University; Seiji Umemoto, Hiroshima University Hospital Center for Comprehensive Medicine; and Atsushi Nagai, Kawasaki Medical University.

Appendix A. Supplementary data

Supplementary data to this article can be found online at https://doi.org/10.1016/j.cct.2021.106449.

References

[1] Ministry of Health, Labour and Welfare, Health Statistics of Japan (Business and Processing Statistics) 2, Trends in Medical Facilities. https://www.mhlw.go.jp/toukei/list/dl/130-28_2.pdf, 2016 (accessed 02 February 2020).
[2] S.M. Yu, Healthy people 2010, Matern. Child Health J. 2 (1998) 63–66, https://doi.org/10.1023/A:1021801927353.
[3] S. Tsuji, T. Murakami, F. Kimura, S. Tanimura, M. Kudo, M. Shozu, et al., Management of secondary infertility following cesarean section: report from the Subcommittee of the Reproductive Endocrinology Committee of the Japan Society of Obstetrics and Gynecology, J. Obstet. Gynaecol. Res. 41 (2015) 1305–1312, https://doi.org/10.1111/jog.12750.
[4] J.M. Dodd, C.A. Crowther, R.M. Grivell, A.R. Deussen, Elective repeat caesarean section versus induction of labour for women with a previous caesarean birth, Cochrane Database Syst. Rev. 7 (2017), CD004906, https://doi.org/10.1002/14651858.CD004906.pub5.
[5] S. Roberge, E. Bujold, Closure of uterus and the risk of uterine rupture, BJOG. 122 (2015) 1542, https://doi.org/10.1111/1471-0528.13095.
[6] S. Sumigama, C. Sugiyama, T. Kotani, H. Hayakawa, A. Inoue, Y. Mano, et al., Uterine sutures at prior caesarean section and placenta accreta in subsequent pregnancy: a case-control study, BJOG. 121 (2014) 866–875, https://doi.org/10.1111/1471-0528.12717.
[7] CAESAR study collaborative group, Caesarean section surgical techniques: a randomised factorial trial (CAESAR), BJOG. 117 (2010) 1366–1376, https://doi.org/10.1111/j.1471-0528.2010.02686.x.
[8] F.G. Cunningham, K.J. Leveno, S.L. Bloom, J.S. Dashe, C.Y. Spong, B.L. Hoffman, et al., Prior Cesarean Delivery, in: Williams Obstetrics, 25th ed., McGraw-Hill, New York, 2018, pp. 591–603.
[9] S. Gabbe, J. Niebyl, H. Galan, Obstetrics: Normal and Problem Pregnancies, 6th ed., Elsevier Saunders, Philadelphia, 2012.
[10] J. Scott, T. Porter, Cesarean delivery, in: R. Gibbs, B. Krian, A. Haney (Eds.), Danforth's Obstetrics and Gynecology, 9th ed., Lippincott Williams & Wilkins, Philadelphia, 2003, pp. 246–252.
[11] Y. Hiramatsu, OGS NOW- Obstetric and Gynecologic Surgery, Cesarean Section Basics and Applications, Total Master, 1st ed., Medical View, Tokyo, 2010, pp. 28–41.
[12] J.A. Greenberg, R.H. Goldman, Barbed suture: a review of the technology and clinical uses in obstetrics and gynecology, Rev. Obstet. Gynecol. 6 (2013) 107–115.
[13] C.C. Chu, J.A. von Fraunhofer, H.P. Greisler, Wound closure biomaterials and devices, CRC Press, Boca Raton, FL, 1997.
[14] Y. Lin, S. Lai, J. Huang, L. Du, The efficacy and safety of knotless barbed sutures in the surgical field: a systematic review and meta-analysis of randomized controlled trials, Sci. Rep. 6 (2017) 23425, https://doi.org/10.1038/srep23425.
[15] R. Angioli, F. Plotti, R. Montera, P. Damiani, C. Terranova, I. Oronzi, et al., A new type of absorbable barbed suture for use in laparoscopic myomectomy, Int. J. Gynaecol. Obstet. 117 (2012) 220–223, https://doi.org/10.1016/j.ijgo.2011.12.023.
[16] F. Alessandri, V. Remorgida, P.L. Venturini, S. Ferrero, Unidirectional barbed suture versus continuous suture with intracorporeal knots in laparoscopic myomectomy: a randomized study, J. Minim. Invasive Gynecol. 17 (2010) 725–729, https://doi.org/10.1016/j.jmig.2010.06.007.
[17] C.C. Chan, C.Y. Lee, Feasibility and safety of absorbable knotless wound closure device in laparoscopic myomectomy, Biomed. Res. Int. (2016), 2849476, https://doi.org/10.26226/morressier.573c1514d462b80296c98dee.
[18] A.Z. Mohamed, U.M. Fouda, K.A. Elsetohy, S.M. Zayed, A.T. Hashem, M.A. Youssef, Barbed sutures versus conventional sutures for uterine closure at cesarean section; a randomized controlled trial, J. Matern. Fetal Neonatal Med. 32 (2019) 710–717, https://doi.org/10.1080/14767058.2017.1388368.
[19] A.W. Chan, J.M. Tetzlaff, D.G. Altman, A. Laupacis, P.C. Gøtzsche, K. Krleža-Jerić, et al., SPIRIT 2013 statement: defining standard protocol items for clinical trials [Japanese], Jpn. Pharmacol. Ther. 45 (2017) 1895–1910.
[20] D. Moher, S. Hopewell, K.F. Schulz, V. Montori, P.C. Gøtzsche, P.J. Devereaux, et al., CONSORT 2010 explanation and elaboration: updated guidelines for reporting parallel group randomised trials, BMJ. 340 (2010) c869, https://doi.org/10.1136/bmj.c869.
[21] S. Maeda, The development research of free software "named randMS" which automatically conducted allocation in a randomized-controlled trial, J. Inst. Stat. Med. Jpn. 1 (2016) 1–9.
[22] STRATAFIX®, Spiral PDS+®. PMDA Interview form. Medical Device Attachment Document Templates. https://www.info.pmda.go.jp/downfiles/md/PDF/340216/340216_22900BZX00123000_A_01_02.pdf, 2020 (accessed 05 February 2020).
[23] Pharmaceuticals and Medical Devices Agency, Vicryl® PMDA Interview form. Medical Device Attachment Document Templates. https://www.info.pmda.go.jp/

downfiles/md/PDF/340216/340216_15700BZY01341000_A_09_02.pdf, 2020 (accessed 05 February 2020).
[24] American College of Obstetricians and Gynecologists: ACOG Practice Bulletin, Clinical management guidelines for obstetrician-gynecologists Number 76, October 2006, postpartum hemorrhage, Obstet. Gynecol. 108 (2006) 1039–1047.
[25] J. Owen, N. Yost, V. Berghella, E. Thom, M. Swain, G.A. Dildy 3rd, et al., Mid-trimester endovaginal sonography in women at high risk for spontaneous preterm birth, JAMA. 286 (2001) 1340–1348, https://doi.org/10.1001/jama.286.11.1340.
[26] Common Terminology Criteria for Adverse Events v5.0 (CTCAE), Harmful Objects Common Language Standard v5.0 Translation of JCOG into Japanese (abbreviated: CTCAE Latest Version-JCOG MedDRA/Jv22.1, September 5, 2019), https://ctep.cancer.gov/protocolDevelopment/electronic_applications/ctc.htm#ctc_50, 2017 (accessed 05 February 2020).
[27] C. Regnard, M. Nosbusch, C. Fellemans, N. Benali, M. van Rysselberghe, P. Barlow, et al., Cesarean section scar evaluation by saline contrast sono- hysterography, Ultrasound Obstet. Gynecol. 23 (2004) 289–292, https://doi.org/10.1002/uog.999.
[28] D. Peleg, R.S. Ahmad, S.L. Warsof, N. Marcus-Braun, Y. Sciaky-Tamir, I. Ben Shachar, A randomized clinical trial of knotless barbed suture vs conventional suture for closure of the uterine incision at cesarean delivery, Am. J. Obstet. Gynecol. 218 (2018) 343.e1–343.e7, https://doi.org/10.1016/j.ajog.2018.01.043.
[29] A. Başbuğ, O. Doğan, A. Ellibeş Kaya, C. Pulatoğlu, M. Çağlar, Does suture material affect uterine scar healing after cesarean section? Results from a randomized controlled trial, J. Investig. Surg. 32 (2019) 763–769, https://doi.org/10.1080/08941939.2018.1458926.
[30] Association of Japan Medical Colleges, Guidance for Management of Institutional Conflict of Interest in Medical and Science Research Institutions. https://www.ajmc.jp/pdf/20190425_02.pdf, 2019 (accessed 28 January 2021).
