# Supplementary material for: Determinants of residual myometrial thickness after cesarean delivery: Comparative analysis of barbed versus conventional sutures—A sub‐analysis from the SPIRAL trial
Source: Int J Gynaecol Obstet. 2025 Jun 5;171(2):861–8. doi: 10.1002/ijgo.70273 (PMC12553111; doi:10.1002/ijgo.70273)
Supplement: Supplementary file 2 — Data S2 [file IJGO-171-861-s004.pdf]

CONSORT 2010 checklist of information to include when reporting a randomised trial*

Checklist about Study Protocol in Contemporary Clinical Trials. 107, (2021): 106449

Section/Topic	Item No	Checklist item	Reported on page No
**Title and abstract**			
	1a	Identification as a randomised trial in the title	P1
	1b	Structured summary of trial design, methods, results, and conclusions (for specific guidance see CONSORT for abstracts)	P2
**Introduction**			
Background and objectives	2a	Scientific background and explanation of rationale	P3
	2b	Specific objectives or hypotheses	P4
**Methods**			
Trial design	3a	Description of trial design (such as parallel, factorial) including allocation ratio	P4
	3b	Important changes to methods after trial commencement (such as eligibility criteria), with reasons	P5-6
Participants	4a	Eligibility criteria for participants	P5
	4b	Settings and locations where the data were collected	P6-7
Interventions	5	The interventions for each group with sufficient details to allow replication, including how and when they were actually administered	P7-11
Outcomes	6a	Completely defined pre-specified primary and secondary outcome measures, including how and when they were assessed	P14
	6b	Any changes to trial outcomes after the trial commenced, with reasons	P15
Sample size	7a	How sample size was determined	P17-18
	7b	When applicable, explanation of any interim analyses and stopping guidelines	P19-20
Randomisation:			
Sequence generation	8a	Method used to generate the random allocation sequence	P8
	8b	Type of randomisation; details of any restriction (such as blocking and block size)	P8
Allocation concealment mechanism	9	Mechanism used to implement the random allocation sequence (such as sequentially numbered containers), describing any steps taken to conceal the sequence until interventions were assigned	P8
Implementation	10	Who generated the random allocation sequence, who enrolled participants, and who assigned participants to interventions	P8

Blinding	11a	If done, who was blinded after assignment to interventions (for example, participants, care providers, those assessing outcomes) and how	P8-9
	11b	If relevant, description of the similarity of interventions	P9
Statistical methods	12a	Statistical methods used to compare groups for primary and secondary outcomes	P19-20
	12b	Methods for additional analyses, such as subgroup analyses and adjusted analyses	P19-20
**Results**			
Participant flow (a diagram is strongly recommended)	13a	For each group, the numbers of participants who were randomly assigned, received intended treatment, and were analysed for the primary outcome	P5-7
	13b	For each group, losses and exclusions after randomisation, together with reasons	P5-7
Recruitment	14a	Dates defining the periods of recruitment and follow-up	P6
	14b	Why the trial ended or was stopped	P12-13
Baseline data	15	A table showing baseline demographic and clinical characteristics for each group	P10
Numbers analysed	16	For each group, number of participants (denominator) included in each analysis and whether the analysis was by original assigned groups	P17-18
Outcomes and estimation	17a	For each primary and secondary outcome, results for each group, and the estimated effect size and its precision (such as 95% confidence interval)	P14-15
	17b	For binary outcomes, presentation of both absolute and relative effect sizes is recommended	P14-15
Ancillary analyses	18	Results of any other analyses performed, including subgroup analyses and adjusted analyses, distinguishing pre-specified from exploratory	
Harms	19	All important harms or unintended effects in each group (for specific guidance see CONSORT for harms)	P12-13
**Discussion**			
Limitations	20	Trial limitations, addressing sources of potential bias, imprecision, and, if relevant, multiplicity of analyses	P21-22
Generalisability	21	Generalisability (external validity, applicability) of the trial findings	P22
Interpretation	22	Interpretation consistent with results, balancing benefits and harms, and considering other relevant evidence	P22
**Other information**			
Registration	23	Registration number and name of trial registry	P3
Protocol	24	Where the full trial protocol can be accessed, if available	P22
Funding	25	Sources of funding and other support (such as supply of drugs), role of funders	P23

*We strongly recommend reading this statement in conjunction with the CONSORT 2010 Explanation and Elaboration for important clarifications on all the items. If relevant, we also recommend reading CONSORT extensions for cluster randomised trials, non-inferiority and equivalence trials, non-pharmacological treatments, herbal interventions, and pragmatic trials. Additional extensions are forthcoming: for those and for up to date references relevant to this checklist, see www.consort-statement.org.
