# Supplementary material for: Determinants of residual myometrial thickness after cesarean delivery: Comparative analysis of barbed versus conventional sutures—A sub‐analysis from the SPIRAL trial
Source: Int J Gynaecol Obstet. 2025 Jun 5;171(2):861–8. doi: 10.1002/ijgo.70273 (PMC12553111; doi:10.1002/ijgo.70273)
Supplement: Supplementary file 4 — Data S4 [file IJGO-171-861-s005.pdf]

Okayama University

1-1-1 Tsushima-naka Kita-ku,Okayama-shi 700-8530, Japan

November 12, 2020

Okayama University Certified Review Board, Ethics Committee

Address of the IRB:
2-5-1 Shikata-cho, Kita-ku, Okayama 700-8558, Japan

Address of the doctor who applied for the IRB approval:
MAKI Jota
Department of Obstetrics & Gynecology Okayama University Graduate School of Medicine.
Dentistry and Pharmaceutical Sciences.
Okayama University Hospital
2-5-1 Shikata-cho, Kita-ku, Okayama 700-8558, Japan

Project Number: jRCT1062200001 (please quote this number with all correspondence)

Assessment of prevention with Spiral to the complications associated with cesarean section wounds -Randomized controlled trial with conventional suture thread- (Prevention of caesarean section scar syndrome using spiral thread)

Funding: Clinical research incentive funds at the our hospital are applicable. (Internal funds)

Dear MAKI Jota ,

During the ethics committee meeting held on March 30, 2020, the project with the above named title was discussed.

The ethics committee consisting of the following members:
MAEDA Yoshinobu, MATSUOKA Kenichi, TAIRA Naruto, KATAOKA Hitomi, MAEKAWA Kenji, ARIYOSHI Noritaka, NAGAI Atsushi, OTOMO Takanobu, UMEMOTO Seiji, YAMAMOTO Yasuyo, ARIMOTO Kohei, AWAYA Tsuyoshi HAYASHI Nobuko, KAWADA Naoko
assembled together and came to the following vote,

The ethics committee approved the planned research project.

Sincerely,

Chairperson MAEDA Yoshinobu, M.D., Ph. D.
Okayama University Certified Review Board
