# Supplementary material for: Determinants of residual myometrial thickness after cesarean delivery: Comparative analysis of barbed versus conventional sutures—A sub‐analysis from the SPIRAL trial
Source: Int J Gynaecol Obstet. 2025 Jun 5;171(2):861–8. doi: 10.1002/ijgo.70273 (PMC12553111; doi:10.1002/ijgo.70273)
Supplement: Supplementary file 5 — Data S5 [file IJGO-171-861-s001.pdf]

Top

Japanese

Date of registration	May. 01, 2020
Last modified on	May. 13, 2020
Trial ID	jRCT1062200001

Scientific Title	Assesment of prevention with Spiral to the complications associated with cesarean section wounds -Randomized controlled trial with conventional suture thread- (Prevention of caesarean section scar syndrome using spiral thread)
Public Title	SPIRAL trial (SPIRAL trial)

Contact for Scientific Queries	Name	Maki Jota
Affiliation	Okayama University Hospital	
Address	2-5-1 Shikata-cho kita-ku, Okayama	
Telephone	+81-86-235-7320	
E-mail	jotajjjo@gmail.com	
Contact for Public Queries	Name	Maki Jota
Affiliation	Okayama University Hospital	
Address	2-5-1 Shikata-cho kita-ku, Okayama	
Telephone	+81-86-235-7320	
E-mail	jotajjjo@gmail.com	

Recruitment status	Recruiting

Anticipated date of first enrollment	May. 01, 2020	
Actual date of first enrollment	May. 07, 2020	
Target sample size	200	
Study Type	Interventional	
Study Design	allocation	randomized controlled trial
masking	open(masking not used)	
control	active control	

**assignment**	parallel assignment	
**purpose**	treatment purpose	
**Key inclusion & exclusion criteria**	**Inclusion Criteria**	For those that meet all the following criteria.<br>(1) A person who is 20 years of age or older when obtaining consent.<br>(2) The person that a document agreement was provided after enough understanding and have been received enough explanation on participating of this study.<br>(3) Patients who are scheduled to undergo selective cesarean section who are hospitalized or admitted to a research institution including Okayama University Hospital Obstetrics and Gynecology on March 31, 2022 after approval of jRCT. An initial cesarean section is a patient who undergoes a cesarean section for the first time because of a breech (pelvic position), fetal bad condition, maternal bad condition,prolonged delivery.<br>(4) Person who can approve that the researcher obtains information on the infant from the medical record.
**Exclusion Criteria**	For those that meet one of the following exclusion criteria.<br>1 She is a changing hospital planned patient on the way<br>2 Patient of the following pregnancy<br>A case to Patients with the following combined pregnancy, to pregnancy with blood disorders,<br>to pregnancy with coagulopathy, to multiple pregnancy,<br>to mental illness complications,<br>to incision other than lower uterine transection,<br>to pre-placental case,<br>to postpartum hemoreage (bleeding more than 2000ml),<br>to emergency cesarean section after full opening of the uterine ostium,<br>to anterior adenomyosis of the anterior wall,<br>to have myoma within the incision.<br>3 Patients who are hypersensitive to constituent metals such as stainless steel or chromium and nickel or triclo-asid.<br>4 In addition, Patients who were judged by the investigator to be inappropriate as study subjects (understood poor understanding / objection of study)	
**Age Minimum**	20age 0month 0week old over	
**Age Maximum**	No limit	
**Gender**	Female	
**Health Condition(s) or Problem(s) Studied**	Selective Ceasearian section	
**Intervention(s)**	Transect the lower uterus about 10cm, and perform uterine suture using two vicrye threads or two spiral plus thread at the timing of uterine repair after delivery. Surgical procedures should be standardized at all facilities. After the principal investigator goes to each facility and practices the procedure in advance, a procedure manual is created and the procedure is unified at each medical institution. Sutures may not be used for longer than 6 weeks and may not be used in areas where sutures need to be spliced or where long fusion times are required, such as fascia. The uterus does not fall in that location.	
**Health Condition(s) Keyword**	The presence of a cesarean scar defect and diverticulum, Selective Ceasearian section	
**Intervention(s) Keyword**	The presence of a cesarean scar defect and diverticulum, Selective Ceasearian section	
**Health Condition(s) Code**	D011248	
**Intervention(s) Code**	D011248	
**Primary Outcome(s)**	Compare the degree of thinning of the scar.<br>The most important evaluation things are transvaginal ultrasonography 6-7 months after surgery, and the position of the uterus (forward or backward) and the thickness of the myometrial wound in the following 1 to 4 were measured and analyzed It.<br>The evaluation of the degree of thinning is based on four measurements of the transvaginal ultrasonic sagittal slice and the thinned portion of the cross section<br>1 Head-to-caudal distance 2 Depth of recess 3 Thymus layer thickness left on the serosa from the most depressed recess 4 Left and right width of recess.<br>The percentage of the remaining myometrium thickness 3 is calculated from the total myometrium thickness 2 and 3 , and the thinning rate is recorded. In addition, 1-4 will be compared between the two groups.	
**Secondary Outcome(s)**	1 Comparison of operation time and required time (time from delivery to completion of uterine suture), bleeding, number of additional Z sutures to stop bleeding in addition to continuous suture, complications during surgery, postoperative infection,years of experience of surgeons<br>2 Child: Age, sex, birth weight, height, umbilical cord blood gas analysis (pH), infant umbilical cord blood t	

est: PO2, PCO2, BE, HCO3-, glucose level, lactate, presence of newborn abnormality
3 Maternal: Age, maternal complications, number of weeks of delivery, opening of uterine ostium (cm), degree of spread (%) physique, BMI, weight gain, treatment content and perinatal period, 1 month postoperative examination Presence or absence of blood retention, the incidence of abnormalities during the treatment and perinatal period were as follows: at discharge, 1 month and 3 months after surgery, transvaginal ultrasonography, hematoma in front of the uterine wound (hematoma) The presence or absence of hematoma is judged to be hematoma if a hematoma is indicated by transluminal ultrasonography.) The incidence of postoperative fever (postoperative fever is a cause of Patients treated with antibiotics other than clinical path as suspected intraperitoneal infection are judged to have fever), presence of dysmenorrhea at 6 or 7 months, presence of chronic pelvic pain (confirmed by interview)
4 Safety ,presence or absence of serious adverse events.

**Primary Sponsor**	

**Source of Monetary Support / Secondary Sponsor**	
**Secondary Sponsor**	

**Source of Monetary Support**	
**Secondary Sponsor**	

**Name of Certified Review Board**	Okayama University Certified Review Board
**Address**	2-5-1 shikata-cho, Kita-ku, Okayama
**Telephone**	+81-86-235-7133
**E-Mail**	ouh-crrb@adm.okayama-u.ac.jp
**Approval Status**	Approval
**Date of approval**	Mar. 30, 2020

**Plan to share IPD**	
**Plan description**	

**Secondary ID(s)**	
**Issuing Authority**	

**Countries of Recruitment（Except Japan）**	none

History of Changes

No	Publication date		
5	Jan. 18, 2024	Detail	Changes
4	June. 02, 2023	Detail	Changes
3	July. 01, 2021	Detail	Changes
2	May. 13, 2020	(this page)	Changes
1	May. 01, 2020	Detail	

List of change history Close

For inqueries here（webadmin-jrct@niph.go.jp）
Click **here** for the privacy policy (only Japanese)

Copyright © National Institute of Public Health, All Rights Reserved.
