# Supplementary material for: Determinants of residual myometrial thickness after cesarean delivery: Comparative analysis of barbed versus conventional sutures—A sub‐analysis from the SPIRAL trial
Source: Int J Gynaecol Obstet. 2025 Jun 5;171(2):861–8. doi: 10.1002/ijgo.70273 (PMC12553111; doi:10.1002/ijgo.70273)
Supplement: Supplementary file 6 — Table S1: [file IJGO-171-861-s002.docx]

**Supplemental File 6**

**Table: “Factors affecting surgical outcomes: multivariate analysis of institutional and surgeon characteristics"**

| ➀Niche length (mm) | | | | | | | | | |
| --- | --- | --- | --- | --- | --- | --- | --- | --- | --- |
| Source | Sum of Squares | | df | | | Mean Square | | F-value | p-value |
| Cervical dilation (≧6cm) | 1.343 | | 1.000 | | | 1.343 | | 0.435 | 0.510 |
| Suture type | 48.539 | | 1.000 | | | 48.539 | | 15.730 | <0.001 |
| Interaction | 0.941 | | 1.000 | | | 0.941 | | 0.305 | 0.581 |
| Error | 666.533 | | 216.000 | | | 3.086 | | - | - |
|  | | | | | | | | | |
| Clinical experience | 1.566 | | 1.000 | | | 1.566 | | 0.509 | 0.476 |
| Suture type | 102.943 | | 1.000 | | | 102.943 | | 33.486 | <0.001 |
| Interaction | 3.224 | | 1.000 | | | 3.224 | | 1.049 | 0.307 |
| Error | 664.041 | | 216.000 | | | 3.074 | | - | - |
|  | | | | | | | | | |
| Facility | 98.444 | | 3.000 | | | 32.815 | | 12.433 | <0.001 |
| Suture type | 63.530 | | 1.000 | | | 63.530 | | 24.069 | <0.001 |
| Interaction | 13.063 | | 3.000 | | | 4.354 | | 1.650 | 0.179 |
| Error | 559.559 | | 212.000 | | | 2.639 | | - | - |
|  | | | | | | | | | |
| ②Niche depth (mm) | | | | | | | | | |
| Source | Sum of Squares | | df | | | Mean Square | | F Value | P |
| Cervical dilation (≧6cm) | 0.249 | | 1.000 | | | 0.249 | | 0.168 | 0.682 |
| Suture type | 20.457 | | 1.000 | | | 20.457 | | 13.809 | <0.001 |
| Interaction | 1.101 | | 1.000 | | | 1.101 | | 0.743 | 0.390 |
| Error | 319.991 | | 216.000 | | | 1.481 | | - | - |
|  | | | | | | | | | |
| Clinical experience | 0.746 | | 1.000 | | | 0.746 | | 0.504 | 0.479 |
| Suture type | 45.842 | | 1.000 | | | 45.842 | | 30.922 | <0.001 |
| Interaction | 0.337 | | 1.000 | | | 0.337 | | 0.228 | 0.634 |
| Error | 320.223 | | 216.000 | | | 1.483 | | - | - |
|  | | | | | | | | | |
| Facility | 1.142 | | 3.000 | | | 0.381 | | 0.255 | 0.857 |
| Suture | 28.180 | | 1.000 | | | 28.180 | | 18.906 | <0.001 |
| Interaction | 3.466 | | 3.000 | | | 1.155 | | 0.775 | 0.509 |
| Error | 315.996 | | 212.000 | | | 1.491 | | - | - |
|  | |  | |  |  | |  | | |
| ③RMT (mm) | | | | | | | | | |
| Source | Sum of Squares | | df | | | Mean Square | | F Value | P |
| Cervical dilation (≧6cm) | 6.004 | | 1.000 | | | 6.004 | | 1.544 | 0.215 |
| Suture type | 67.317 | | 1.000 | | | 67.317 | | 17.317 | <0.001 |
| Interaction | 0.175 | | 1.000 | | | 0.175 | | 0.045 | 0.832 |
| Error | 839.680 | | 216.000 | | | 3.887 | | - | - |
|  | | | | | | | | | |
| Clinical experience | 20.835 | | 1.000 | | | 20.835 | | 5.496 | 0.020 |
| Suture type | 104.841 | | 1.000 | | | 104.841 | | 27.654 | <0.001 |
| Interaction | 5.813 | | 1.000 | | | 5.813 | | 1.533 | 0.217 |
| Error | 818.884 | | 216.000 | | | 3.791 | | - | - |
|  | | | | | | | | | |
| Facility | 76.271 | | 3.000 | | | 25.424 | | 8.103 | <0.001 |
| Suture type | 32.220 | | 1.000 | | | 32.220 | | 10.270 | 0.002 |
| Interaction | 86.727 | | 3.000 | | | 28.909 | | 9.214 | <0.001 |
| Error | 665.125 | | 212.000 | | | 3.137 | | - | - |
|  | | | | | | | | | |
| ④Niche width (mm) | | | | | | | | | |
| Source | Sum of Squares | | df | | | Mean Square | | F Value | P |
| Cervical dilation (≧6cm) | 1.608 | | 1.000 | | | 1.608 | | 0.245 | 0.621 |
| Suture type | 53.100 | | 1.000 | | | 53.100 | | 8.076 | 0.005 |
| Interaction | 0.012 | | 1.000 | | | 0.012 | | 0.002 | 0.966 |
| Error | 1420.271 | | 216.000 | | | 6.575 | | - | - |
|  | | | | | | | | | |
| Clinical experience | 8.105 | | 1.000 | | | 8.105 | | 1.238 | 0.267 |
| Suture type | 87.183 | | 1.000 | | | 87.183 | | 13.321 | <0.001 |
| Interaction | 0.115 | | 1.000 | | | 0.115 | | 0.018 | 0.895 |
| Error | 1413.701 | | 216.000 | | | 6.545 | | - | - |
|  | | | | | | | | | |
| Facility | 55.112 | | 3.000 | | | 18.371 | | 2.997 | 0.032 |
| Suture type | 45.283 | | 1.000 | | | 45.283 | | 7.388 | 0.007 |
| Interaction | 44.358 | | 3.000 | | | 14.786 | | 2.412 | 0.068 |
| Error | 1299.417 | | 212.000 | | | 6.129 | | - | - |
|  | | | | | | | | | |
| ^a^Rate of niche (%) | | | | | | | | | |
| Source | Sum of Squares | | df | | | Mean Square | | F Value | P |
| Cervical dilation (≧6cm) | 1.717 | | 1.000 | | | 1.717 | | 0.013 | 0.910 |
| Suture type | 3011.094 | | 1.000 | | | 3011.094 | | 22.510 | <0.001 |
| Interaction | 74.393 | | 1.000 | | | 74.393 | | 0.556 | 0.457 |
| Error | 28893.970 | | 216.000 | | | 133.768 | |  |  |

| Clinical experience | 263.379 | 1.000 | 263.379 | 1.991 | 0.160 |
| --- | --- | --- | --- | --- | --- |
| Suture type | 6193.812 | 1.000 | 6193.812 | 46.831 | <0.001 |
| Interaction | 134.650 | 1.000 | 134.650 | 1.018 | 0.314 |
| Error | 28567.607 | 216.000 | 132.257 | - | - |
|  | | | | | |
| Facility | 648.296 | 3.000 | 216.099 | 1.725 | 0.163 |
| Suture type | 2886.347 | 1.000 | 2886.347 | 23.034 | <0.001 |
| Interaction | 1468.147 | 3.000 | 489.382 | 3.905 | 0.010 |
| Error | 26565.467 | 212.000 | 125.309 | - | - |
|  | | | | | |

Abbreviation: df, degrees of freedom, RMT, residual myometrial thickness.

Notes:

Analysis performed using two-way ANOVA.

Rate of niche calculated as (niche depth/[niche depth + residual myometrial thickness]) × 100.

Statistical significance set at P<0.05.

Dash (-) indicates not applicable.
